# Estimating health service utilization potential using the supply-concentric demand-accumulation spatial availability index: a pulmonary rehabilitation case study

**DOI:** 10.1186/s12942-020-00224-2

**Published:** 2020-08-03

**Authors:** Kevin A. Matthews, Anne H. Gaglioti, James B. Holt, Anne G. Wheaton, Janet B. Croft

**Affiliations:** 1 Centers for Disease Control and Prevention, Atlanta, GA USA; 2 National Center for Primary Care, Morehouse School of Medicine, Atlanta, GA USA

## Abstract

The potential for a population at a given location to utilize a health service can be estimated using a newly developed measure called the supply-concentric demand accumulation (SCDA) spatial availability index. Spatial availability is the amount of demand at the given location that can be satisfied by the supply of services at a facility, after discounting the intervening demand among other populations that are located nearer to a facility location than the given population location. This differs from spatial accessibility measures which treat absolute distance or travel time as the factor that impedes utilization. The SCDA is illustrated using pulmonary rehabilitation (PR), which is a treatment for people with chronic obstructive pulmonary disease (COPD). The spatial availability of PR was estimated for each Census block group in Georgia using the 1105 residents who utilized one of 45 PR facilities located in or around Georgia. Data was provided by the Centers for Medicare & Medicaid Services. The geographic patterns of the SCDA spatial availability index and the two-step floating catchment area (2SFCA) spatial accessibility index were compared with the observed PR utilization rate using bivariate local indicators of spatial association. The SCDA index was more associated with PR utilization (Morans I = 0.607, P < 0.001) than was the 2SFCA (Morans I = 0.321, P < 0.001). These results suggest that the measures of spatial availability may be a better way to estimate the health care utilization potential than measures of spatial accessibility.

## Introduction

The potential for a population at a given location to utilize a health service can be estimated as the spatial availability of a service. Spatial availability is the amount of demand at the given location that can be satisfied by the supply of services at a facility, after discounting the intervening demand among other populations that are located nearer to a facility location than the given population location. This differs from spatial accessibility measures, which treat absolute distance or travel time as the primary factor that impedes people from using a health care service [1–6]. While distances or travel times from demand locations to supply locations is a common way of measuring impedance, [7–9] the demand for the service among populations that reside closer to the available health care facilities has never been investigated as a source of impedance. Formally, we define the spatial availability of a health care service at a given population location(i) as the amount of demand at that can be satisfied by the supply of services at a facility(j), after discounting the intervening demand among other populations (ii) that are located nearer to a facility location(j) than the given population location(i).

Here, we introduce the supply-concentric demand-accumulation (SCDA) spatial availability index as new approach for estimating utilization potential. We illustrate the SCDA using pulmonary rehabilitation (PR) in Georgia. PR is an effective treatment for chronic obstructive pulmonary disease (COPD), which is an irreversible respiratory disease that worsens over time. Improving the availability of PR can potentially improve the lives of over 15 million Americans with COPD [10]. We chose Georgia because it is within a region of the United States that has significantly higher COPD prevalence, Medicare hospitalizations for COPD, and COPD-related mortality than other areas in the United States [10, 11]. PR is a multi-modal intervention; a typical session may include breathing exercises, education on disease processes and physiology, psychological support, nutrition counseling, peer support, and exercise training [12]. Patients with COPD who participate in PR have better exercise outcomes, fewer chronic comorbidities, and a higher quality of life [13]. PR programs usually last from 8 to 12 weeks, with 2 or 3 sessions per week [14]. Given the time intensity and frequency of this treatment, adherence to a prescribed regimen may be hindered or facilitated by the amount of demand for PR among a population with COPD that can be satisfied by the number of treatments that are available at their nearby PR facilities.

In this study, we compare the geographic pattern of spatial availability of PR using the SCDA spatial availability index with the geographic pattern of a contemporary measure of spatial accessibility called the two-step floating catchment area (2SFCA) spatial accessibility index [15]. Then we compared both measures of health service utilization potential with the geographic pattern of observed PR utilization. While we used PR to illustrate the SCDA spatial availability index, this method could be used to estimate the utilization potential of any specific procedure in a healthcare utilization database that contains locational information about each health care facility and the number of services they provide.

### Data

The Centers for Medicare & Medicaid Services (CMS) annually publishes 100% Medicare Limited Data Set (LDS)–Outpatient Files [16]. This data set contains all Fee-for-Service (FFS) claims submitted by institutional outpatient facilities. The analytic cohort consists of Medicare FFS beneficiaries aged ≥ 65 years who resided in Georgia in 2014 and were treated for COPD with PR using Healthcare Common Procedure Coding System (HCPCS) code G0424. A medical diagnosis of a chronic respiratory condition including chronic bronchitis (ICD-9-CM codes 491.0–491.1), obstructive chronic bronchitis, without exacerbation (ICD-9-CM code 491.20), other chronic bronchitis (ICD-9-CM code 491.8), other emphysema (ICD-9-CM code 492.8), or chronic airway obstruction, not elsewhere classified (ICD-9-CM code 496) is required for reimbursement under this HCPCS code. Since PR typically requires several treatments to be fully effective, each patient receives multiple PR treatments.

PR facilities were defined as any facility used by the analytic cohort. One important characteristic of the LDS data is that the geographic detail for the beneficiaries is low (e.g., county of residence), but the geographic detail about the provider is high (e.g. street address of practice location). That is, the LDS data provides the National Provider Inventory (NPI) number of each provider which, when matched to the publicly available NPI database, contains the full street address of their practice location.

Any facility located within states that border Georgia were included if they billed Medicare for services provided to a Georgia resident. Multiple providers can practice at a single facility and multiple facilities can be located within a single ZIP Code. The supply locations in this study were the geometric centroid of the ZIP Code tabulation area (ZCTA) corresponding to the ZIP Code of their practice location in their National Provider Inventory (NPI) record. Then, the number of services for the providers and facilities were summed together if they had the same ZIP Code. For example, if ten providers with the same ZIP Code each performed ten services, the total supply at the ZCTA would be equal to 100.

### Calculating the estimated demand field

One necessary input for calculating the SCDA spatial availability index is the estimated demand field, which is a pre-computed estimate of demand for PR at each population location [17]. We estimated demand for PR among Medicare Fee-for-Service Medicare (FFS) beneficiaries aged ≥ 65 years who were diagnosed with COPD at each Census block group. The geographic and population data were collected by US Census Bureau as part of the 2010 decennial Census. Geographic and age-specific population data for each Census 2010 block group were downloaded from the National Historical Geographic Information System database [18]. Demand estimates were needed because the demand for PR is higher than the observed utilization among the analytic cohort. That is, not all people who needed PR (e.g., persons with COPD) used it. For this study, demand for PR was estimated for each Census block group in Georgia (n = 5529) and in block groups located within the counties of other states that border of Georgia (n = 5576). Two publicly available datasets published by CMS were used to create the estimated demand field. The first is a county-level dataset containing the prevalence of selected chronic conditions (including COPD) for Medicare beneficiaries enrolled in the Fee-for-Service (FFS) program [19]. Beneficiaries with COPD were identified if a patient had at least one inpatient, skilled nursing facility, home health agency, or two carrier claims with any International Classification Diseases, 9th edition Clinical Modification (ICD-9-CM) codes 490–492 or 496 present on any claim within a 1  year reference period beginning in 2014 [20]. The second is a county-level dataset containing the percent of Medicare beneficiaries who were FFS beneficiaries, which was necessary given that only 66% of the US population aged ≥ 65 years are FFS enrollees; this percentage varies substantially across the United States [21].

Equation 1 shows that the estimated demand (E_i_) for block group (i) was calculated by multiplying the 2010 Census population of persons aged ≥ 65 years (P_i_) at block group(i) by the county-level percentage of that population who were Fee-for-Service beneficiaries (FFS_ci_) and then by the county-level percentage of those FFS beneficiaries who were diagnosed with COPD (COPD_ci_).

1 $$E_{i} = P_{i} * COPD_{ci} * FFS_{ci}$$

where, i = index of block groups, c = index of counties in state of Georgia, E_i_ = the estimated number of Medicare FFS beneficiaries aged ≥ 65 years diagnosed with COPD at block group i, P_i_ = the number of people aged ≥ 65 years residing within block group i, FFS_ci_ = County-level percentage of population aged ≥ 65 years who were Medicare Fee-for-Service enrollees in 2014, COPD_ci_ = County-level percentage of Medicare FFS beneficiaries aged ≥ 65 years diagnosed with chronic obstructive pulmonary disease (COPD) in county c.

### Overview of the supply-concentric demand accumulation (SCDA) spatial availability index

The most commonly used contemporary measure of spatial accessibility is called the two-step floating catchment area (2SFCA) spatial accessibility index [22]. The 2SFCA spatial accessibility index uses two types of floating catchments. Floating catchments are areas drawn around a location and have been defined in a number of ways, such as by a fixed Euclidean distance, [23–25] or travel time from a population location to a facility location [15, 26]. An alternate approach is for all catchments to vary in size according to some threshold value, such as the number of people needed to support the facility [5, 17, 27]. The first type of catchment is centered on the facilities where the supply of a service is located. A provider-to-population (P2P) ratio is calculated for each facility using the number of providers at the facility as the numerator and the number of people who reside within the facility’s catchment as the denominator. The second type of catchment is centered on each population location. The final 2SFCA measure for a given population location is calculated in step by summing the P2P ratios for all health care facilities located within the floating catchment of that given population location. Another important advancement was the creation of the enhanced 2SFCA (2SFCA) spatial accessibility index, which uses discrete distance zones to account for the decreasing service utilization potential among the population of an area as their distance or travel time from facilities increased [15]. However, this distance decay parameter can also be estimated continuously using a variety of functions; in this study we used a Gaussian function thus removing the need for discrete zones [4].

The SCDA spatial availability index also uses floating catchments but uses them in an entirely different way than the 2SFCA. The SDCA only produces floating catchment areas around facility locations, but it produces as many catchment areas as there are population locations—or as many population locations located within a threshold distance or travel time if a threshold is imposed by the researcher. The “supply-concentric” component refers to the concentric catchment areas that surround each facility as the distance or travel time from the facility to each population location increases. These concentric catchments will be circular if based on Euclidean distance and oddly shaped if based on travel time along a road network. The Network Analyst extension of ArcGIS 10.5.1 (ESRI, Redlands, CA) and ESRI Streetmap data were used to create an origin(i)-destination (j) matrix of travel time in minutes from each PR facility to each population-weighted Census block group centroid. Unique facility(j)-population location(i) dyads are created and denoted as SCDA_ji_ when the facility(j) catchment intersects each population location(i). The “demand accumulation” component refers to the intervening demand for the health service that accumulates as the distance or travel time from the facility to each population location increases.

The SCDA spatial availability index (SCDA_i_) requires two general steps. The first step is to calculate the SCDA ratio for each SCDA_ji_ dyad. Equation 2 shows that the numerator for a given dyad is the number of services observed at the facility location(j) and the accumulated demand at each population location(i) is the denominator. The accumulated demand at a given population location(i) is the estimated demand (Eq. 1) at that location plus the sum of the estimated demand at all population locations (ii) that were located nearer to a facility(j). An SCDA_ji_ ratio > 1 indicates that the observed number of services at facility(j) exceeds the accumulated demand at population location(i) and that the supply at facility(j) can fully satisfy the accumulated demand at each population location(i). An SCDA ratio < 1 indicates that the accumulated demand at population location(i) exceeds the supply at facility(j).

2 $$SCDA_{ji} Ratio = \frac{{O_{j} }}{{\sum E_{i} \in \left( {d_{jii} \le d_{ji} } \right) }}$$

where, SCDA_ji_ = Facility(j)-specific SCDA ratio at population location(i), j = index of facility location, i = index of population locations, O_j_ = observed number of procedures at facility location j, Ei = the estimated number of Medicare FFS beneficiaries aged ≥ 65 years diagnosed with COPD at block group i, d_ji_ = Travel time from facility location(j) to population location(i), d_jii_ = Travel time from facility location(j) to population location (ii).

The SCDA spatial availability index for each population location(i) is a summary measure of the SCDA ratios for all facilities (j) within a threshold distance (d_0_) from the population location(i). Eq. 3 shows that the SCDA availability index for each population location(i) is the gravity-weighted mean of all SCDA_ji_ ratios of facilities within a threshold distance (d_0_) from the population location(i). The numerator is the observed number of services provided at each facility(j) weighted by a distance decay weight G(d_ji_,d_0_) presented in Eq. 4 [4]. This parameter is used to account for the decay in utilization potential as travel time increases. The denominator attributed to each population location is the accumulated demand (E_i_) for PR divided by the number of PR facilities within a 60-min travel time (N_j_); the denominator is not gravity weighted because the demand for PR from a person with COPD is independent of whether or not they are able to access the service. Logarithmic transformation aids in interpretation of the SCDA spatial availability index. An SCDA spatial availability index > 1, or log(SCDA index) > 0, at a population location(i) indicates the number of services at all facilities within 60 min can fully satisfy the accumulated demand at population location(i). An SCDA index < 1, or log (SCDA_ij_) < 0, at a population location(i) indicates that the supply of the services at all facilities within 60 min is unable to satisfy the accumulated demand at population location(i).

3 $$SCDA_{i} = \log \left( {\frac{{\mathop \sum \nolimits_{i} O_{j} *G\left( {d_{ji} ,d_{0} } \right)}}{{\left( {\frac{{\sum E_{i} \in \left( {d_{jii} \le d_{ji} } \right)}}{{N_{j} < d_{0} }}} \right)}}} \right)$$

where, i = index of population locations, j = index of facility location, C_ji_ = Facility(j)-specific SCDA ratios at population location(i), sorted by d_ji._ d_ji_ = Travel time from facility location(j) to population location(i), d_jii_ = Travel time from facility location(j) to population location(ii), C_i_ = The sum of the facility(j)-specific SCDA ratios of all facilities at block group(i) within a 60-min travel time, d_0_ = Threshold travel time (60 min), G(d_ji_, d_0_) = Distance decay weight based on the Gaussian function, N_j_ = number of facilities within threshold travel time.

4 $$G\left( {d_{ji} ,d_{0} } \right) = \left\{ {\begin{array}{*{20}c} {\frac{{e^{{{ - 1/2* \left( {{\raise0.7ex\hbox{${d_{ji} }$} \!\mathord{\left/ {\vphantom {{d_{ji} } {d_{0} }}}\right.\kern-0pt} \!\lower0.7ex\hbox{${d_{0} }$}}} \right)^{2} }}} - e^{ - 1/2} }}{{ - e^{ - 1/2} }} \quad d_{ji} \le d_{0} } \\ {0 d_{ji} > d_{0} } \\ \end{array} } \right.$$

where, d_ji_ = Travel time from facility location(j) to population location(i), G(d_ji_, d_0_) = Distance decay weight based on the Gaussian function, d_0_ = Threshold travel time.

To ensure comparability between the SCDA and 2SFCA, we used the same demand estimates from Eq. 1, the same number of PR treatments observed at each facility location(j), and the same Gaussian distance decay function. We also used travel time along a road network and imposed a 60-min travel time limit (d_0_). We used the equation for the two-step floating catchment area (2SFCA) spatial accessibility index which can be found elsewhere [15]. Block groups > 60 min from a facility were symbolized as their own map class, but they were assigned the minimum value of the block group with the longest travel time that was within 60 min.

We used the bivariate local Moran’s I statistic to evaluate the spatial association of the SCDA spatial availability index and the 2SFCA spatial accessibility index; this statistic measures the degree of positive or negative linear association between the value for one variable at a given location and the average of another variable at neighboring locations [28]. We also used Pearson’s R correlation to measure the association between the two measures of utilization potential and the observed PR utilization rate for all block groups in Georgia. The PR utilization rate for each county used the total number of PR procedures observed in the county in the numerator and the total number of beneficiaries who received PR as the denominator. However, the SCDA index and the 2SFCA index were measured at the block group level while the PR utilization rate was a county-level measure because the most detailed level of geography for beneficiaries in the Medicare Limited Data Set (LDS)–Outpatient Files is county. Calculating measures of association under these conditions is generally known as the change of spatial support problem (COSP) where spatial support refers to the shape, size, and orientation of the geographic units into which spatial measurements are taken [29]. We addressed the COSP by transforming the PR utilization variable from a county-level variable to a block group-level using a process called downscaling. We downscaled each county’s PR utilization rate by assigning its value to all block groups nested within that county. We also calculated these correlation coefficients stratified by metropolitan status using the 2013 NCHS Urban–Rural Classifications Scheme for Counties [30]. This scheme breaks counties in the United States into 6 classes, which we further collapsed into the following three categories: (1) large central and fringe metropolitan, (2) medium and small metropolitan, and (3) micropolitan and noncore.

We used ArcGIS 10.5 for spatial data handling and cartography. We used the ArcGIS Network Analyst to create an origin–destination matrix of travel times along a street network consisting of 45 PR facilities(j) in and around the state of Georgia to the 11,305 block groups(i) in Georgia and counties that neighbor Georgia for a total of 508,725 unique facility(j)-population location(i) dyads. However, we only show results for the 5529 block groups within Georgia. We used custom scripts written for STATA 15/SE to calculate the SCDA index, the Two-Step Floating Catchment Area (2SFCA) index, the PR utilization rate, and Pearson’s R correlation coefficients. Geoda 1.14.0 was used to calculate the bivariate local Moran’s I statistic for each block group.

## Results

**Fig. 1:**
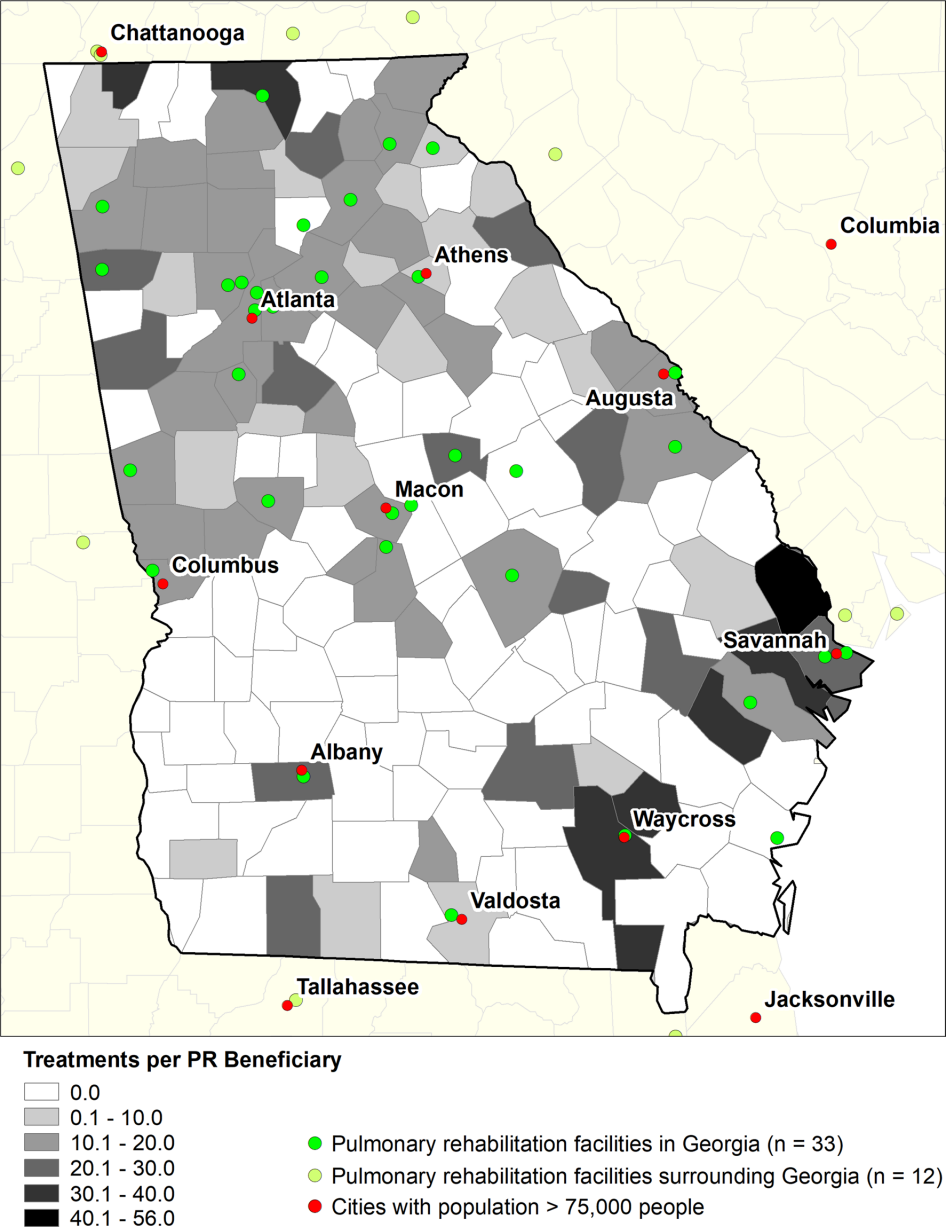
Pulmonary rehabilitation utilization rate and locations of facilities used by Georgia beneficiaries, 2014

In 2014, 1105 Medicare FFS beneficiaries aged ≥ 65 years who resided in Georgia received a total of 18,166 PR treatments at the 33 PR facilities practicing in Georgia or at one of the 12 PR facilities located outside the state. PR facilities were in only 18.9% (n = 30) of the 159 counties in Georgia. Almost half of all counties had at least one beneficiary who obtained PR in 2014 (n = 80). In 2010 population data, there were 985,137 persons aged ≥ 65 years. Of the 5529 block groups in Georgia, only 3.4% were more than 60 min away from a PR provider representing 3.6% of the population aged ≥ 65 years. The county-level PR utilization rate ranged from 0 to 56 treatments per PR beneficiary (Fig. 1). Most counties that had a PR utilization rate > 0 were in the northern half of Georgia, which is where most of the PR facilities were located.

**Fig. 2:**
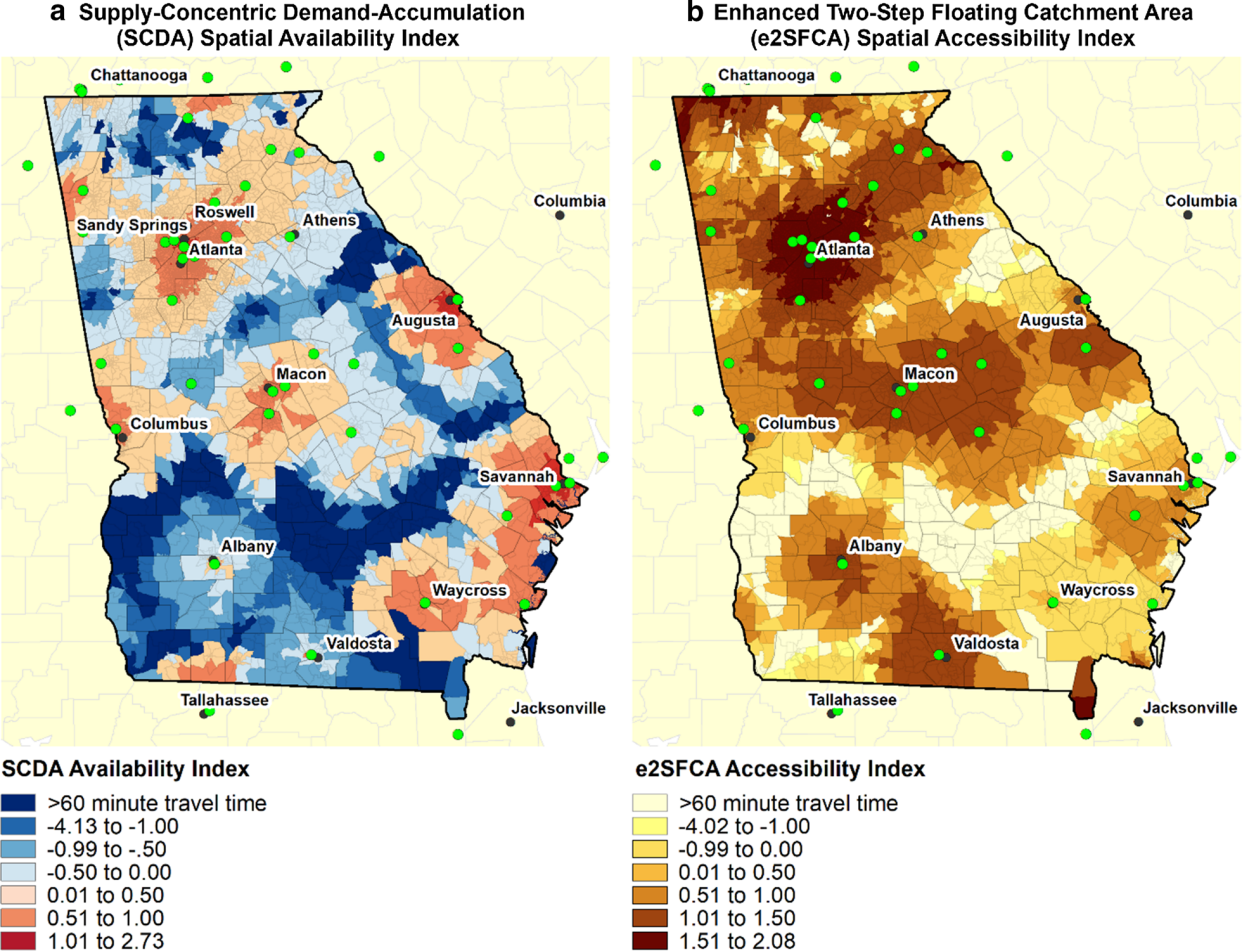
**a** The spatial availability of pulmonary rehabilitation (PR) and (**b**) spatial accessibility of PR in Georgia, 2014

The association between the geographic pattern of the PR utilization rate (Fig. 1) and the SCDA index (Fig. 2a) was relatively high using both the Pearson’s R (aspatial) and local Moran’s I (spatial) measures of association (R = 0.692 and I = 0.607, *P *< 0.001). The association of the PR utilization rate and the 2SFCA index (Fig. 2b) was much lower (R = 0.268 and I = 0.321, *P *< 0.001). The SCDA index was more strongly associated with the PR utilization rate than the 2SFCA index even when stratified by rural–urban status. Note that we did not conduct the local Moran’s I tests by urban–rural status because the observations need to be spatially contiguous. In the large central and fringe metropolitan areas, the SCDA was more associated with PR utilization (R = 0.589, *P *< 0.001) than was the 2SFCA (R = 0.442, *P *< 0.001). The association between the SCDA index and PR utilization was highest in the medium and small metropolitan counties (R = 0.690, *P *< 0.001), but this urban–rural category exhibited the lowest association for the 2SFCA (R = 0.264), *P *< 0.001). PR utilization was more correlated in the nonmetropolitan counties with the SCDA spatial availability index (R = 0.431, *P *< 0.001) than with the 2SFCA spatial accessibility index (R = 0. 327, *P *< 0.001).

The association between the geographic pattern of the SCDA index (Fig. 2a) and the 2SFCA index (Fig. 2b) was relatively low using both the Pearson’s R (aspatial) and local Moran’s I (spatial) measures of association (R = 0.433 and I = 0.524, *P *< 0.001). The association was strongest in the large central and fringe metropolitan counties (R = 0.910, *P *< 0.001) and in the non-metropolitan counties (R = 0.846, *P *< 0.001), but was much smaller in the small and medium metropolitan areas (R = 0.143, *P *< 0.001).

**Fig. 3:**
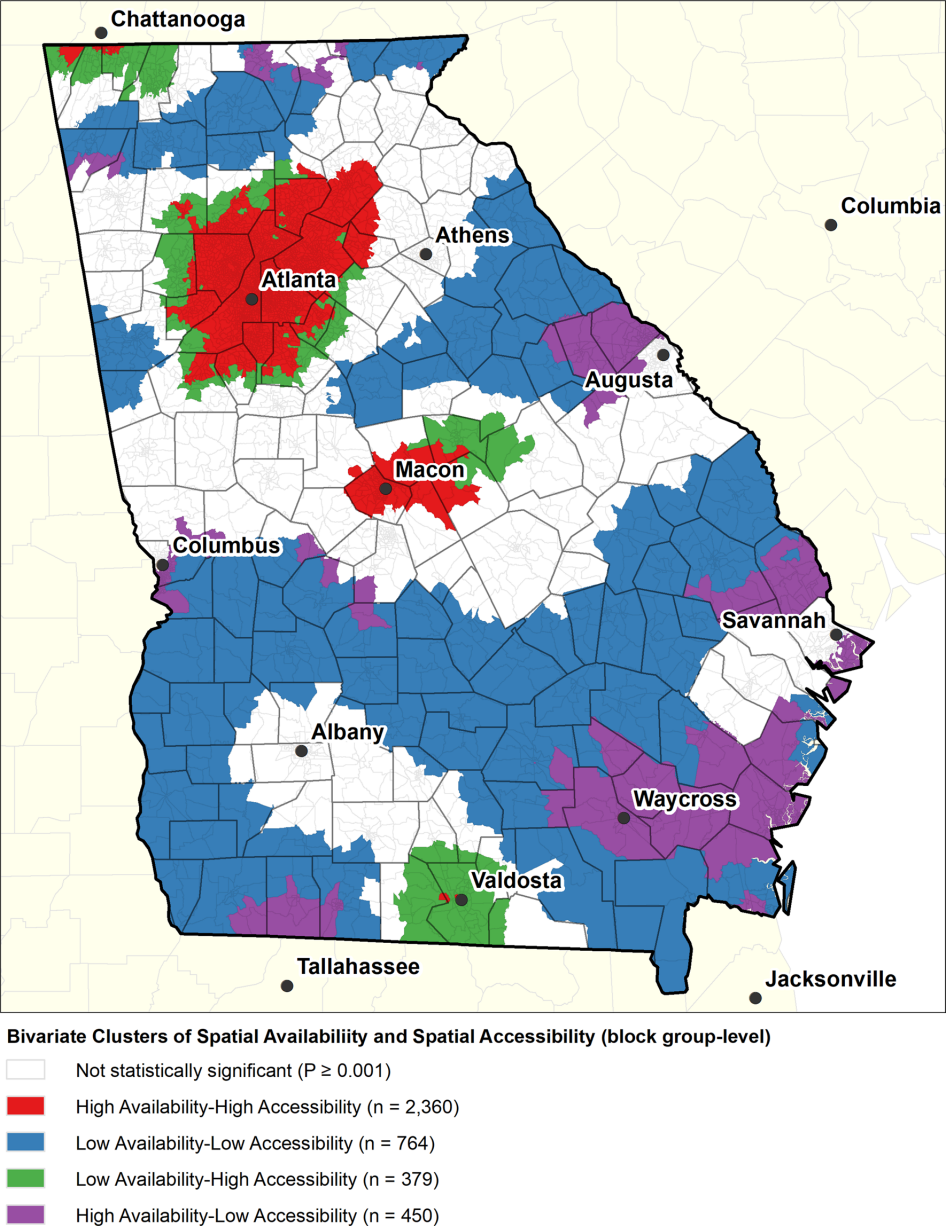
Co-location of the Supply-Concentric Demand Accumulation (SCDA) spatial availability index and the Two-Step Floating Catchment Area (2SFCA) spatial accessibility index

The spatial availability map (Fig. 2a) shows the geographic distribution of PR availability. Areas in red indicate that the supply of the services at all facilities within 60 min is potentially able to satisfy the demand for PR services while areas in blue have insufficient levels of supply to satisfy the demand. The spatial accessibility map (Fig. 2b) shows the sum of the facility-to-population ratios for providers located within 60 min of a block group. Despite the general pattern of high spatial availability and accessibility in block groups surround the PR facility locations, the geographic patterns of the two measures of potential utilization are quite different. For example, the SCDA in the block groups around Albany show a relatively few block groups with enough availability to satisfy the demand. But, according to the 2SFCA map, spatial accessibility to PR remains relatively high even a great distance from Albany. Likewise, the SCDA measure is much more concentrated around the provider locations than the 2SFCA measure. For example, Fig. 2b shows a large swath of relatively high spatial accessibility to PR in the middle of the state (around Macon) and another around Valdosta, but these large areas do not appear on Fig. 2a. Figure 3 highlights where the two measures are most similar and where they are different. Overall, it highlights that the high values of spatial accessibility do not dissipate as quickly with distance as the spatial availability measure suggesting that the spatial accessibility measure may overestimate the utilization potential of those populations. For example, the swath of red surround Atlanta shows where accessibility and availability are both relatively high, but there is a large fringe of green where there is high accessibility, but low availability. This phenomenon also occurs near Macon, Valdosta, and the block groups on the northern border near Chattanooga, TN. This suggests that the availability is exhausted at much shorter travel times. The purple areas represent places with high availability but low accessibility. These areas tend to be more rural so that a relatively small supply at a location can potentially satisfy a relatively large rural area.

## Discussion

In this study, we introduced the SCDA spatial availability index as a new measure of health service utilization potential and compared its results with those of the commonly used 2SFCA spatial accessibility index. We found that that the spatial availability of pulmonary rehabilitation (PR) in Georgia was significantly associated with the spatial accessibility of PR, but the spatial accessibility index appears to overestimate PR utilization potential. This overestimation may also explain why the SCDA measure of PR spatial availability is more highly correlated with PR utilization than the measure of PR spatial accessibility. While we measured the spatial availability to PR facilities to Medicare beneficiaries in the state of Georgia, the method can be generalized to any given health service in any part of the world that has data about the location and supply of that service and a population who needs it.

Like the 2SFCA and other measures of spatial accessibility, the SCDA spatial availability index also models supply and demand simultaneously, which is considered an essential property of any measure of spatial utilization potential [7–9]. However, this study also highlights the importance of *intervening demand* as the primary factor impeding utilization of a health service at a given population location(i). The SCDA method presented here also builds upon the field-based framework for creating spatially adaptive floating catchment (SAFC) areas, which relies on pre-computed estimates of demand for a health service at each population location [17]. It is important to note the relationship between the concentric catchments described in this paper and the SAFCs; while each facility has several SCDAs, the one where the supply first exceeds the demand is the SAFC for a given facility.

One of the strengths of the SCDA index compared to the 2SFCA is in its interpretability. While the value of the 2SFCA continually increases from its minimum value to suggest greater accessibility, it does not detect whether an area has an adequate supply of a service. The SCDA values center around an equilibrium point where demand equals supply thus explicitly describes which areas have enough supply to satisfy demand (SCDA > 0) and which areas do not (SCDA < 0). Another strength of this study is that we were able to measure spatial availability and accessibility along the border of Georgia by using any facility used by Georgia beneficiaries, including those located outside the state and the field-based framework of pre-computed estimates of demand for a health service at each population location. Another strength of the SCDA is the SCDA index is sensitive to the number of procedures that were provided rather than the number of clinicians providing the service. This is important because estimates of availability may be biased if they are based only on types of providers or facilities at a location [31]. This approach also addresses a phenomenon where a majority of physicians practice at multiple facility sites [32] because the supply is based on the number of patients seen at a facility rather than by which provider specifically treated the patient.

There are also a few limitations that are specific to this study. First, the LDS data only contained 1 year of data about beneficiaries aged ≥ 65 years who were enrolled in Fee-for-Service programs and used an institutional facility for PR. This means that we had a relatively small number of beneficiaries to calculate utilization rates and the observed number of PR services for each facility. Furthermore, the CMS data did not have information about beneficiaries who obtained PR using managed care plans, Medicare Advantage programs, or at a Veteran’s Affairs clinic or hospital. This limitation does not affect the SCDA method or the comparisons we made within the context of the data that were available, but it is possible that the data is not representative of the full landscape of PR utilization in Georgia. Second, the highest level of geographic detail for Medicare beneficiaries is their county of residence, which required us to downscale the observed PR utilization rates to the census block group-level. While the correlation coefficients are not as robust as they would be if all measures were originally at the block group-level, these findings still suggest that the spatial availability measure is potentially a better estimate of utilization potential than the 2SFCA method. However, there is another limitation of the SCDA that does not apply to the 2SFCA. One property of the SCDA is that the geographic reach of the supply at two or more individual facilities located within the same geographic unit will be much smaller than the reach of a single location with the supply data aggregated together. Our solution was to aggregate the supply data for two or more facilities located within the same ZIP Code and repositioned the facility locations to one statistically central location. The 2SFCA excels over SCDA because it produces a more geographically refined measure of utilization potential because the supply data does not need to be aggregated and repositioned.

This SCDA method has wide application beyond one region in the United States or the therapeutic procedure known as pulmonary rehabilitation. While we used pulmonary rehabilitation to illustrate the SCDA spatial availability index, this method could be used to measure any specific procedure in a healthcare utilization database that contains locational information about each health care facility and the number of services it provided to a defined population. National health systems that maintain complete records of each patient, the type of care they sought, and the location where care was delivered are best positioned to take full advantage of this method. However, even health systems that do not maintain robust datasets can still take advantage of this method as long they have data about the locations of the available service facilities and the locations of the populations that potentially need the service.
